# ITGBL1 transcriptionally inhibited by JDP2 promotes the development of pancreatic cancer through the TGF-beta/Smad pathway

**DOI:** 10.1590/1414-431X2022e11989

**Published:** 2022-05-16

**Authors:** Tiancong Du, Ke Zhang, Zhongbo Zhang, Aijia Guo, Guilin Yu, Yuanhong Xu

**Affiliations:** 1 Department of Pancreatic and Biliary Surgery, The First Hospital of China Medical University, Shenyang, Liaoning, China; 2 Department of Anorectal Surgery, Panjin Central Hospital, Panjin, Liaoning, China

**Keywords:** Pancreatic cancer, ITGBL1, JDP2, TGF-*β* signaling pathway

## Abstract

Pancreatic cancer (PC) is one of the malignant tumors with the worst prognosis worldwide because of a lack of early diagnostic markers and efficient therapies. Integrin, beta-like 1 (ITGBL1) is a *β*-integrin-related extracellular matrix protein and is reported to promote progression of some types of cancer. Nevertheless, the function of ITGBL1 in PC is still not clear. Herein, we found that ITGBL1 was highly expressed in PC tissues compared to normal tissues (P<0.05) and PC patients with higher TGBL1 expression showed worse prognosis. PANC-1 and AsPC-1 cells were used for gain/loss-of-function experiments. We found that ITGBL1-silenced cells exhibited decreased proliferation, migration, and invasion abilities and delayed cell cycle, whereas ITGBL1 overexpression reversed these malignant behaviors. ITGBL1 was also demonstrated to activate the TGF-*β*/Smad pathway, a key signaling pathway in PC progression. Additionally, ITGBL1 expression was found to be suppressed by a suppressor of PC progression, c-Jun dimerization protein 2 (JDP2). Results of dual-luciferase assay indicated that transcription factor JDP2 could inhibit TGBL1 promoter activity. ITGBL1 overexpression inversed the effects of JDP2 up-regulation on cell function. Collectively, we concluded that ITGBL1 may be transcriptionally suppressed by JDP2 and promote PC progression through the TGF-*β*/Smad pathway, indicating that ITGBL1 may have therapeutic potential for the treatment of PC.

## Introduction

Pancreatic cancer (PC) is one of the most recalcitrant and fatal tumors, with poor prognosis and low chemotherapy efficacy (1,2). PC patients are usually diagnosed in the late stage, and the five-year survival rate of patients is less than 10% (3,4). Up to now, PC is the seventh leading cause of cancer, but it is predicted to become the second cause of cancer-related deaths by 2030 (5). Most patients with PC often develop metastases (6). Metastasis and delayed diagnosis are the causes of inefficient treatment for PC patients (7). In recent decades, poor prognosis, high mortality, and little improvement in prognosis have prompted us to explore the mechanism of PC and potential therapeutic targets.

Integrin beta-like 1 (ITGBL1) encodes ten tandem integrin EGF-like repeat domain-containing protein (TIED), which was discovered and cloned from an osteoblast cDNA library (8). ITGBL1 has no similarity with most proteins of the EGF-like protein family, having neither an RGD (Arg-Gly-Asp)-binding domain nor a transmembrane domain, indicating that ITGBL1 fulfills a function differing from those of integrin (9). Previous studies have indicated that ITGBL1 plays a key role in some types of cancer. For instance, ITGBL1 was proven to accelerate epithelial-mesenchymal transition (EMT), migration, and invasion by activating the NF-*κ*B signaling pathway in prostate cancer (10). The function of ITGBL1 in gastric cancer was also investigated and the conclusion was that ITGBL1 is a potential predictor by promoting the migration and invasion of gastric cancer cells (11). In PC, however, the expression and underlying molecular mechanism of ITGBL1 remains uncharacterized.

In the current study, we analyzed mRNA expression of ITGBL1 in normal and PC tissues and found that ITGBL1 was highly expressed in PC tissues, and the overall survival rate of patients with high ITGBL1 expression was worse. In addition, the JASPAR website (http://jaspar.genereg.net/) predicted that transcription factor c-Jun dimerization protein 2 (JDP2) could bind to the promoter of ITGBL1. JDP2 was reported to be downregulated in PC tissues and its downregulation is associated with tumor metastasis and poor prognosis in patients with PC (12). Hence, based on the evidence above, we speculated that ITGBL1 might be involved in the development of PC and its expression may be transcriptionally regulated by JDP2.

## Material and Methods

### Database analysis

The Gene Expression Profiling Interactive Analysis (GEPIA) database (http://gepia.cancer-pku.cn/) was used to detect the mRNA level of ITGBL1 in pancreatic adenocarcinoma and normal tissues. The relationship between ITGBL1 expression and prognosis of PC patients was analyzed using the Kaplan-Meier plotter database (http://www.kmplot.com/analysis/index.php?p=background). The database above is web-based tools to deliver fast and customizable functionalities based on TCGA and GTEx data. The binding between JDP2 and *ITGBL1* promoter was predicted by JASPAR website (http://jaspar.genereg.net/).

### Cell culture and transfection

Human PC cells SW1990 were purchased from Procell (China) and cultured in Leibovitz's L-15 medium (Solarbio, China) with 10% FBS. PANC-1, AsPC-1, and BxPC-3 cell lines were purchased from iCell Bioscience (China). PANC-1 cells were treated with 10% FBS in DMEM medium. In addition, RPMI-1640 medium with 10% FBS was used for AsPC-1 and BxPC-3 cells. The above cells were cultured in an incubator at 37°C and 5% CO_2_.

PANC-1 and AsPC-1 cells were used to silence or overexpress ITGBL1. For ITGBL1 downregulation, PANC-1 and AsPC-1 cells were transfected with short hairpin RNAs (shRNAs) specifically targeting ITGBL1 or the negative control (NC) for 48 h. For ITGBL1 overexpression, PANC-1 and AsPC-1 cells were transfected with ITGBL1 overexpression vector or empty vector for 48 h. For co-transfection, PANC-1 cells were co-transfected with ITGBL1 overexpression vector and JDP2 overexpression vector for 24 h. The transfections were mediated by Lipofectamine 3000 (Invitrogen, USA), according to the manufacturer's instruction.

### Quantitative real-time PCR

Total RNA from PC cells was extracted and then reverse-transcribed into complementary DNA (cDNA). Quantitative real-time PCR (qRT-PCR) analysis was carried out using a SYBR Green kit (Solarbio, China), and the obtained data were processed by ExicyclerTM 96 (Bioneer, Korea). Primer sequences used for qRT-PCR were as follows (5′-3′): ITGBL1 F: TGCACCTGCTATCCTCC; ITGBL1 R: CACAATGACAAGAACCCT. Relative fold expressions were calculated with the comparative threshold cycle (2^−ΔΔCt^) method.

### Western blot and antibodies

The concentration of the extracted protein was measured using a BCA protein assay kit (Beyotime, China) according to the manufacturer's protocol. After SDS-polyacrylamide gel electrophoresis, the separated protein was transferred onto polyvinylidene difluoride membranes (Millipore, USA) at 80 V for 1.5 h. The protein was then subjected to immune blot analysis with specific antibodies for ITGBL1 (Abclonal, China), Smad2 (Abclonal), p-Smad2 (Abclonal), Smad3 (Abclonal), p-Smad3 (Abclonal), Smad7 (Abclonal), JDP2 (Affinity, China), TGF-*β*1 (Abclonal), Ki67 (Abclonal), MMP2 (Proteintech, China), and *β*-actin (Santa Cruz, USA). All antibodies were diluted to 1:1000, except JDP2, which was diluted to 1:2000. In addition, the secondary antibody reagent in this study was IgG-HRP (Beyotime, China) with 1:5000 dilutions.

### MTT assay

The PANC-1 and AsPC-1 cells were seeded onto 96-well plates at a density of 4×10^3^ cells/well. After transfection, the cells were incubated at 37°C for 0, 24, 48, 72, and 96 h. Then, MTT assay was performed by the MTT cell proliferation and cytotoxicity assay kit (Beyotime) to detect the cell viability of ITGBL1-silenced cells and ITGBL1-overexpressed cells. For data analysis, we recorded the absorbance at 570 nm by an enzyme-labeled instrument (Biotek, USA).

### EdU assay

The proliferation of PANC-1 and AsPC-1 cells was measured using an EdU cell proliferation detection kit (Imaging, KeyGen Biotech, China). Forty-eight hours after transfection, the preheated EdU staining solution with a final concentration of 10 μM was added to the cells of each group and cultured for 2 h in a cell incubator at 37°C and 5% CO_2_. Afterward, 4% paraformaldehyde was used to fix the cells. After adding 0.5% Triton X-100 in PBS to each well, the cells were incubated at room temperature for 20 min. After washing the cells in PBS twice, Click-iT reaction solution (KeyGen Biotech) was added and then incubated for 30 min at room temperature in the dark. The cells were stained with Hoechst33342 (KeyGen Biotech) for 15 min and washed in PBS. Photographs were obtained under a fluorescence microscope (Olympus, Japan).

### Cell cycle analysis

The cells were cultured in 6-well culture plates. After transfection, PANC-1 and AsPC-1 cells were collected by centrifugation at 90 *g* for 5 min at room temperature. Later, cells were treated with pre-cooled 70% ethanol and fixed overnight. The collected cells were washed twice with PBS, stained with PI/RNase A, and then incubated at ambient temperature for 30 min. Flow cytometry (Agilent, USA) was performed to detect cell cycle at 48 h.

### Wound healing assay

The medium used for PANC-1 and AsPC-1 cells was replaced with a serum-free medium. In quick succession, the transfected cells were treated with 1 μg/mL mitomycin C (Sigma-Aldrich, USA) for 1 h before wounding. After scratching the cell layer with a 200 μL pipette tip, the cells were washed three times with a serum-free medium. Finally, images of migrating cells were recorded at the times of 0 and 24 h, and the wound region of the different samples was assessed and compared.

### Transwell assay

The invasive activity of the cells was tested using transwell chambers (Corning, USA). PANC-1 and AsPC-1 cells were seeded in 24-well plates and incubated for 24 h. Invading cells on the lower surface were fixed with 4% paraformaldehyde for 15 min and stained with 0.5% crystal violet for 5 min. The cells were counted under an optical microscope (200×; Olympus), and 5 random fields were captured to compare the invasive ability of ITGBL1.

### Gelatin zymography assay

Proteins in PC cells were extracted and diluted into the same protein concentration by using a BCA Protein Assay Kit (Beyotime). Samples were separated by 10% SDS-PAGE containing 0.1% gelatin. The gels were washed for 40 min in a washing buffer (50 mM Tris-HCl, 5 mM CaCl_2_, 1 μM ZnCl_2_, pH 7.6) to remove the SDS, and then incubated for 40 h at 37°C in reaction buffer (50 mM Tris-HCl, 5 mM CaCl_2_, 1 μM ZnCl_2_, 0.02% Brij, 0.2 mol/L NaCl). After incubation, the gel was placed in a solution containing 0.05% Coomassie Brilliant Blue G-250, 30% methanol, and 10% ethanoic acid for 3 h. After that, the intensive bands of MMP-2 and MMP-9 were visualized.

### Dual-luciferase assay

The JDP2 overexpression or control vectors were transfected with the pGL3 vectors containing the candidate ITGBL1 promoter sequence (from -2000 bp to +50 bp or -1200 bp to +50 bp) into PANC-1 cells. After 24-h transfection, the cells were examined for the luciferase activity using the luciferase assay kit (KeyGen Biotech). The enzyme-labeled instrument M200Pro (TECAN, Switzerland) was used to investigate the luciferase activity of the ITGBL1 promoter.

### *In vivo* tumorigenesis

The ITGBL1-silenced PANC-1 cells or PANC-1 cells transfected with NC shRNA were injected subcutaneously into nude mice. Five mice were used in each group. Tumor size was evaluated every 4 days. Four weeks later, the mice were sacrificed and tumor tissues were obtained for subsequent experiments.

### Statistical analysis

Statistical analysis was conducted by GraphPad Prism software 8.0 version (USA) and P<0.05 was considered to be statistically significant. When more than two groups were compared, one-way ANOVA was performed. A *t*-test was performed to analyze the difference between two groups. All data are reported as means±SD.

## Results

### ITGBL1 is highly expressed in PC tissues

**Figure 1:**
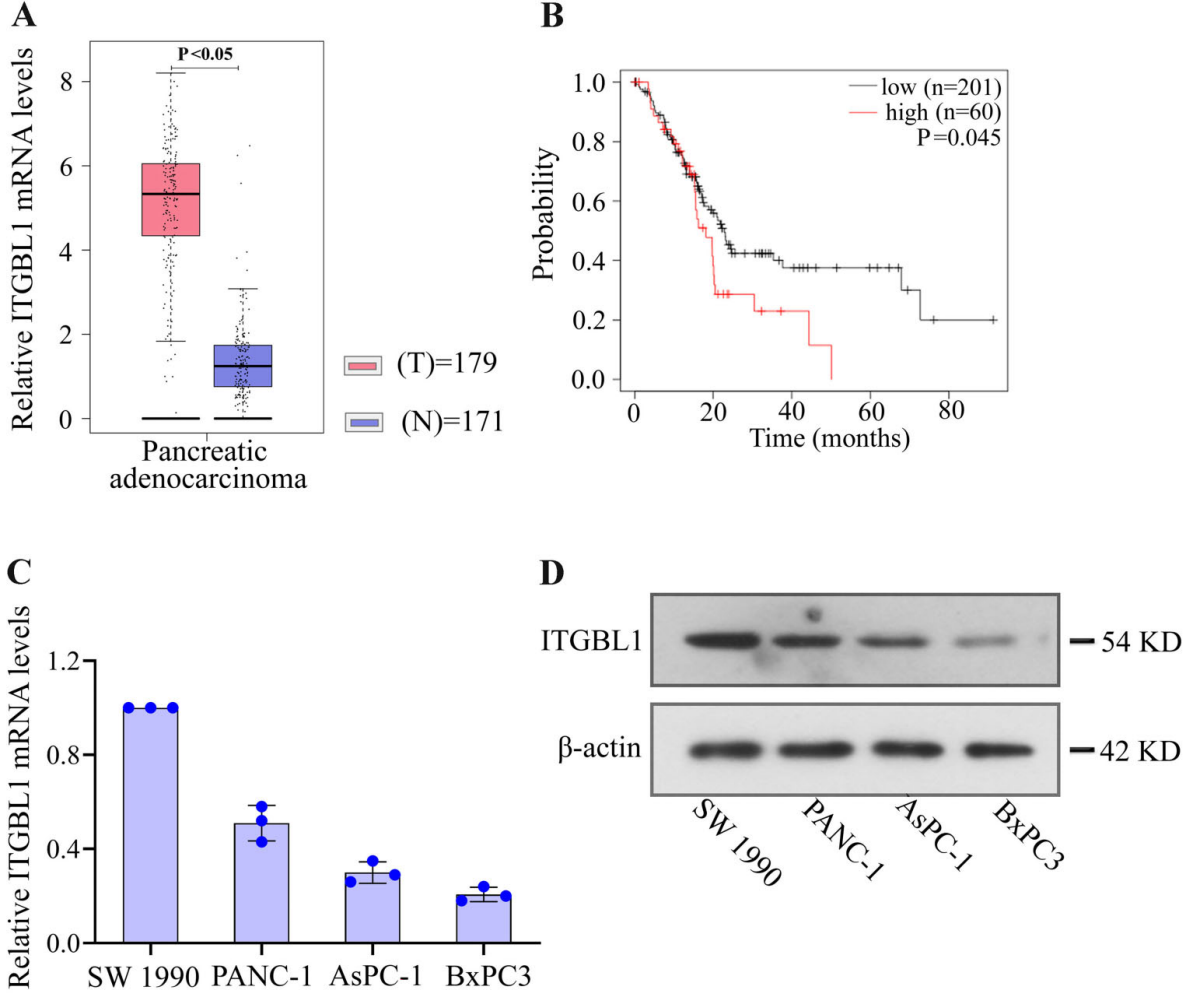
ITGBL1 is highly expressed in pancreatic cancer tissues. **A**, ITGBL1 mRNA expression in pancreatic adenocarcinoma tissues and normal tissues was analyzed by the GEPIA website based on the TCGA database. **B,** The overall survival time of PC patients with ITGBL1 high expression or low expression was analyzed by the Kaplan-Meier plotter database. **C**, Relative ITGBL1 mRNA expression in pancreatic cancer cell lines (SW 1990, PANC-1, AsPC-1, and BxPC3) was detected by real-time quantitative PCR. **D**, Relative ITGBL1 protein expression, standardized by *β*-actin, in pancreatic cancer cell lines (SW 1990, PANC-1, AsPC-1, and BxPC3) was detected by western blot. Data are reported as means±SD for N=3 (ANOVA).

To determine the expression of ITGBL1 in PC tissues, GEPIA database was used to detect the mRNA level of ITGBL1. The results showed that ITGBL1 was up-regulated in PC tissues compared to normal tissues (Figure 1A). Moreover, the relationship between ITGBL1 expression and prognosis of PC patients was analyzed using the Kaplan-Meier plotter database. PC patients with higher ITGBL1 expression had a shorter survival time than those with lower ITGBL1 expression (Figure 1B). The mRNA and protein levels of ITGBL1 in PC cell lines were measured by qRT-PCR and Western blot (Figure 1C and D).

### Overexpression of ITGBL1 facilitated proliferation of PC cells

**Figure 2:**
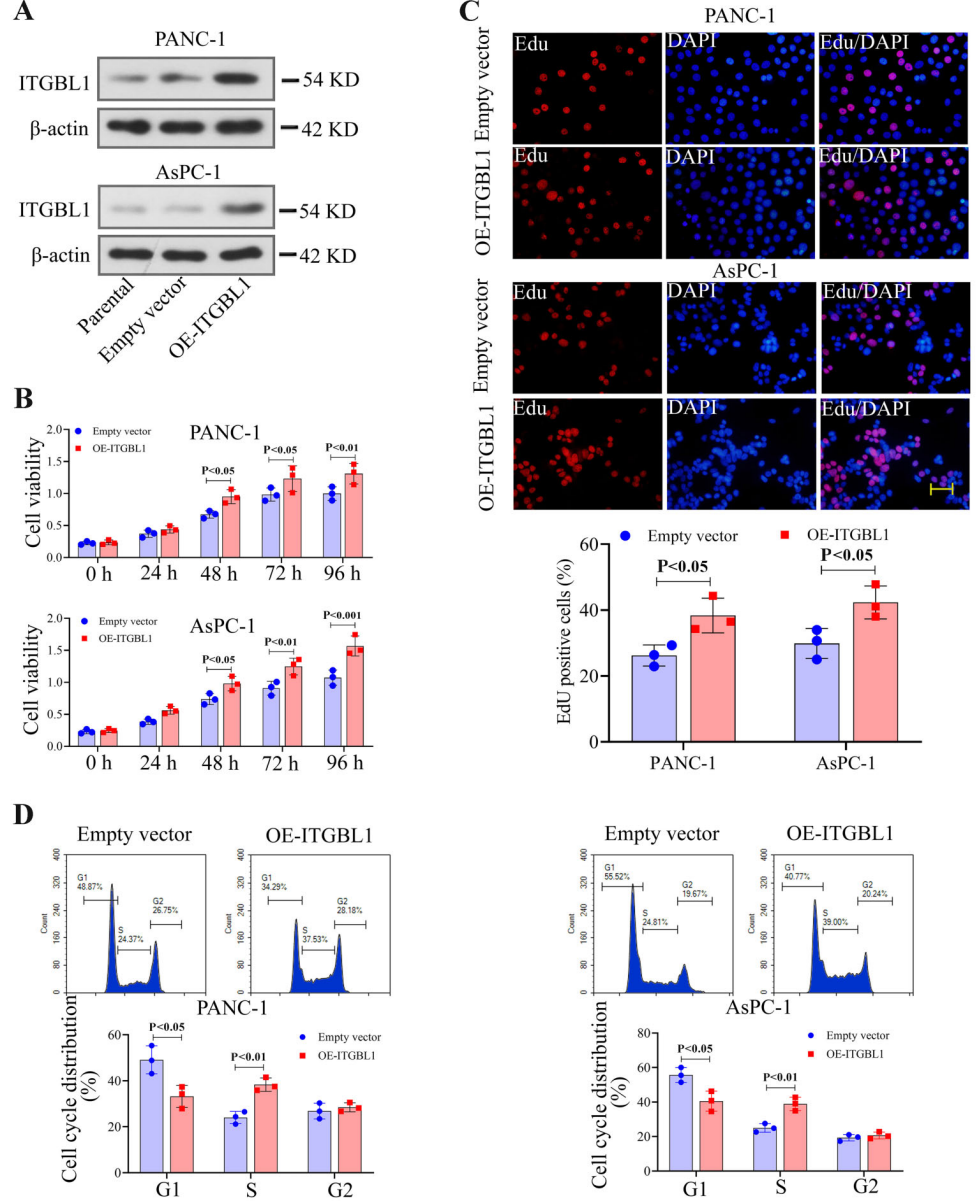
ITGBL1 overexpression (OE-ITGBL1) promoted the proliferation of pancreatic cancer cells. **A**, Relative ITGBL1 protein expression, standardized by *β*-actin, in ITGBL1-overexpressed cells was performed by western blot. **B**, Cell viability of pancreatic cancer cells with ITGBL1 overexpression was detected by MTT assay. **C**, EdU assay was performed on pancreatic cancer cells with ITGBL1 upregulation. Scale bar=50 μm. **D**, Cell cycle distribution of ITGBL1-overexpressed cells was measured by a flow cytometer. Data are reported as means±SD for N=3 (*t*-test).

ITGBL1 overexpression vectors (OE-ITGBL1) were transfected into PANC-1 and AsPC-1 cells to achieve ITGBL1 overexpression. Our results showed that the protein expression of ITGBL1 was elevated in PC cells transfected with OE-ITGBL1 (Figure 2A). As shown in Figure 2B, ITGBL1 overexpression significantly increased cell viability over time, in which a significant increase at 48 h (P<0.05) was observed. Results of the EdU assay also confirmed that the proliferation ability of PC cells was promoted by ITGBL1 overexpression, (Figure 2C) and flow cytometry analysis showed that ITGBL1 overexpression decreased cells in the G1 phase and increased cells in the S phase (Figure 2D).

### ITGBL1 silencing suppressed the proliferation of PC cells

**Figure 3:**
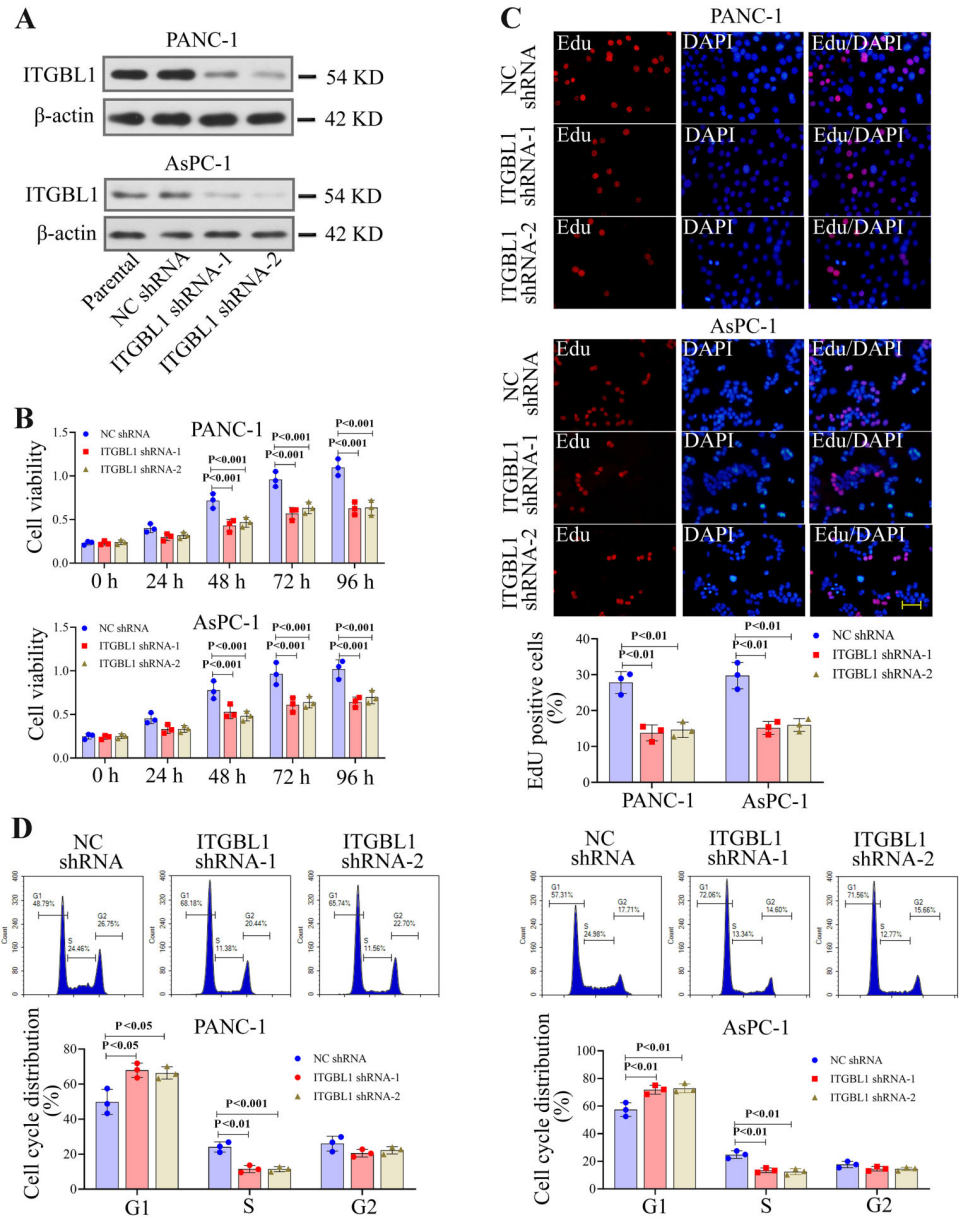
ITGBL1 knockdown inhibited the proliferation of pancreatic cancer cells. **A**, Relative ITGBL1 protein expression, standardized by *β*-actin, in ITGBL1-silenced cells was performed by western blot. **B**, Cell viability of ITGBL1-silenced pancreatic cancer cells was detected by MTT assay. **C**, EdU assay was performed on pancreatic cancer cells with ITGBL1 downregulation. Scale bar=50 μm. **D**, Cell cycle distribution of ITGBL1-silenced cells was measured by a flow cytometer. Data are reported as means±SD for N=3 (ANOVA). NC: negative control.

ITGBL1 silencing was achieved by transfecting PC cells with shRNAs specifically targeting ITGBL1. The knockdown efficiency was verified by Western blot (Figure 3A). ITGBL1 silencing significantly decreased the viability of PC cells over time (Figure 3B). Consistently, the decreased multiplication capacity of ITGBL1-silenced cells was verified by the EdU assay (Figure 3C). In addition, ITGBL1 inhibition arrested cells in the G1 phase and decreased cells in the S phase (Figure 3D).

### ITGBL1 facilitated the migration and invasion of PC cells *in vitro*

**Figure 4:**
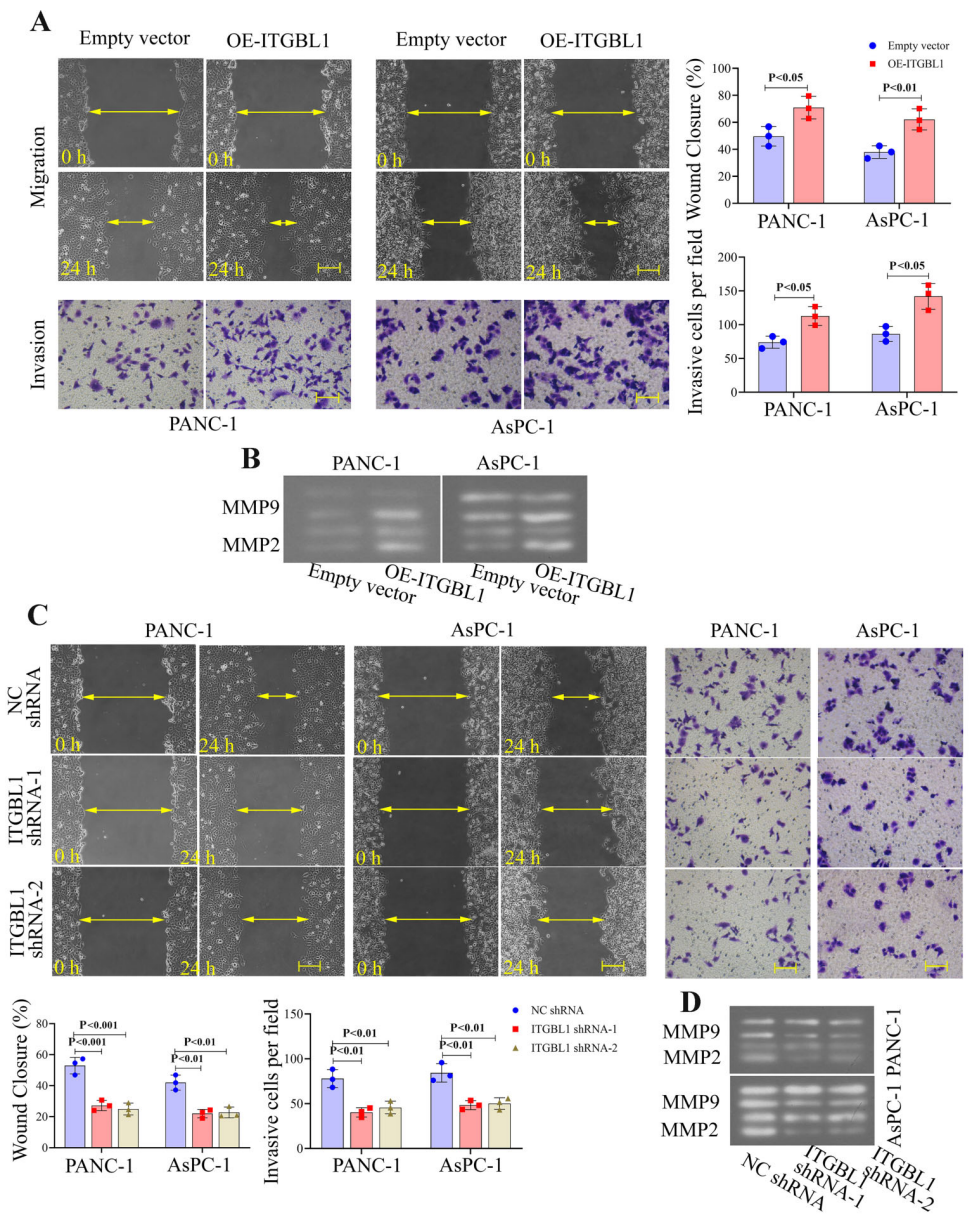
ITGBL1 promoted the migration and invasion of pancreatic cancer cells **A** and **C**, Cell migration and invasion in ITGBL1-overexpressed (OE-ITGBL1) or silenced cells were evaluated by wound healing (scale bar=200 μm) and transwell invasion (scale bar=100 μm) assays, respectively. **B** and **D**, The activities of MMP2 and MMP9 in the supernatant of ITGBL1-overexpressed or silenced cells were measured by gelatin zymography assay. Data are reported as means±SD for N=3 (ANOVA).

ITGBL1 overexpression vector transfection significantly increased the invasion and migration capacities, whereas ITGBL1 inhibition had the opposite effects (Figure 4A and C). The expression of related markers was also measured by gelatin zymography assay. The results showed that the activities of matrix metallopeptidase 2 (MMP-2) and matrix metallopeptidase 9 (MMP-9) were increased by ITGBL1 overexpression and reduced by ITGBL1 knockdown (Figure 4B and D).

### ITGBL1 activated the TGF-*β*/Smad pathway in PC cells

**Figure 5:**
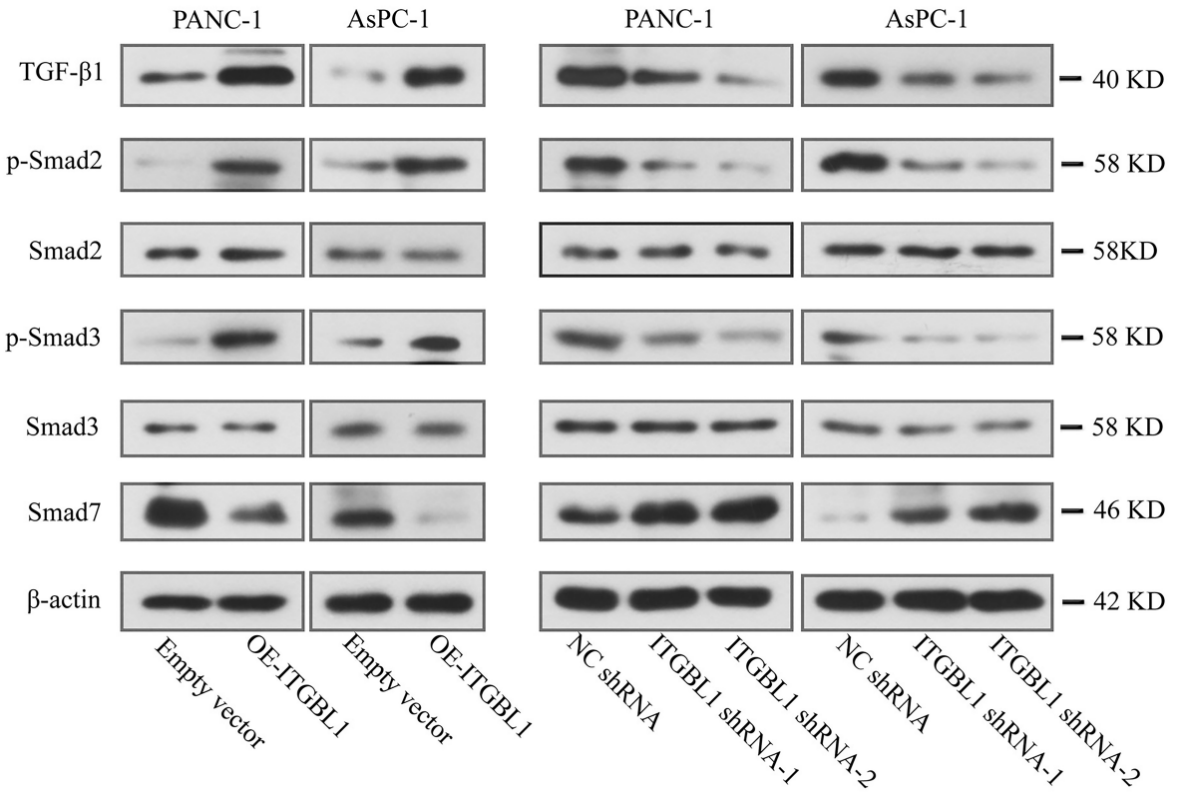
ITGBL1 activated the TGF-*β*/Smad pathway in pancreatic cancer cells. The protein expression levels of TGF‐*β*1, Smad2, p-Smad2 (S465/S467), Smad3, p-Smad3 (S423/S425), and Smad7 in pancreatic cancer cells with ITGBL1 upregulation (OE-ITGBL1) or downregulation were detected by western blot. N=3. NC: negative control.

We further explored the potential mechanism of ITGBL1 involved in the development of PC. The results of Western blot showed that TGF‐*β*1, p-Smad2, and p-Smad3 were increased in ITGBL1-overexpressed cells, while Smad7 protein presented the opposite status by comparing with the empty vector (Figure 5). ITGBL1 knockdown had the opposite effect on the expression of these proteins.

### ITGBL1 was inhibited by JDP2 in PC cells and mediated the effects of JDP2 overexpression

**Figure 6:**
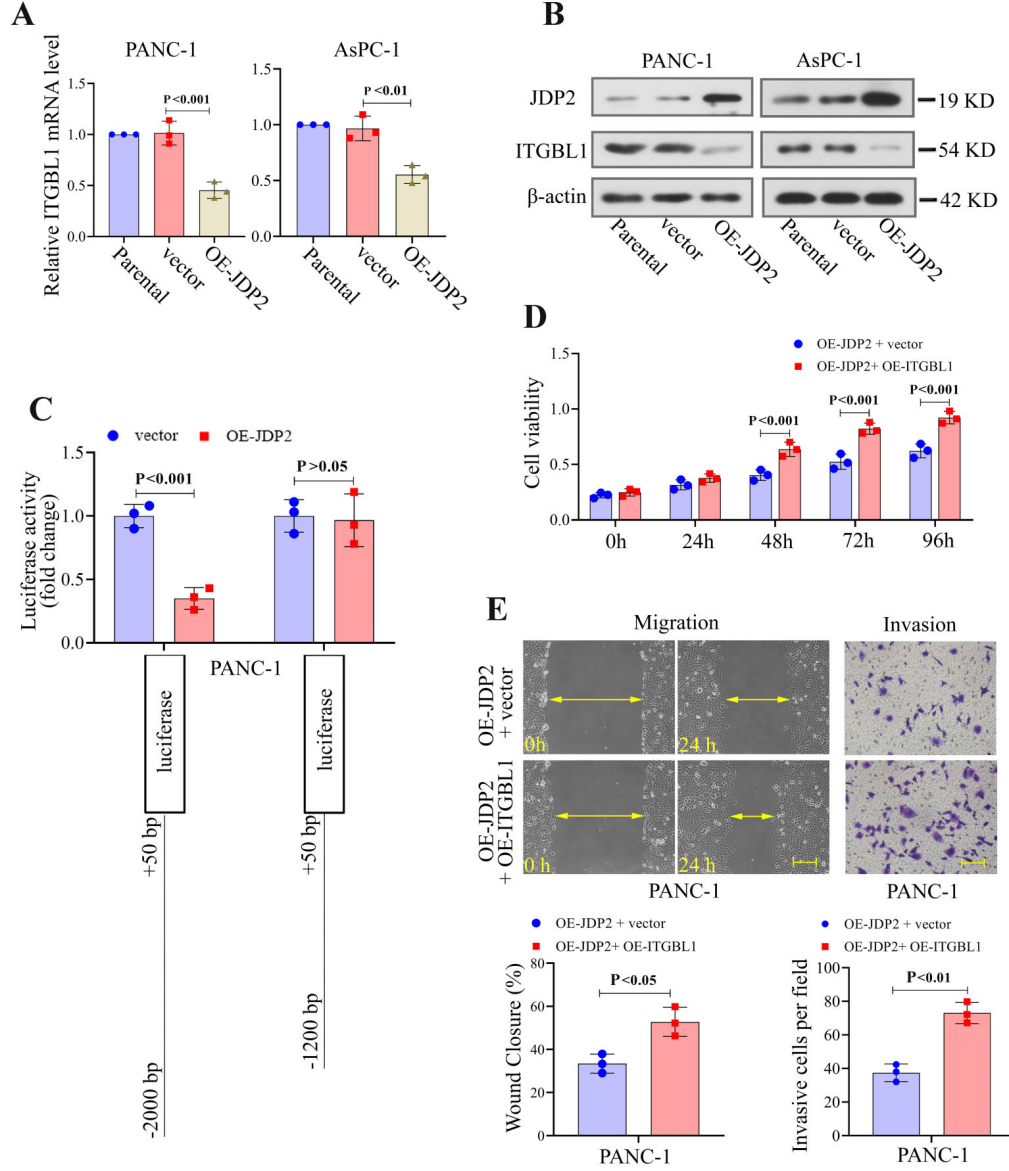
ITGBL1 was transcriptionally inhibited by JDP2 in pancreatic cancer cells and mediated the effects of JDP2 overexpression (OE). **A,** ITGBL1 mRNA levels in JDP2-overexpressed cells were measured by real-time quantitative PCR. **B,** JDP2 and ITGBL1 protein levels in pancreatic cancer cells with JDP2 overexpression were measured by western blot. **C,** Luciferase activity was detected after PANC-1 cells were co-transfected with a luciferase reporter vector and JDP2 overexpression vector or empty vector. **D** and **E**, Cell viability, migration (Scale bar=200 μm), and invasion (Scale bar=100 μm) of JDP2-overexpressed cells transfected with ITGBL1 overexpression vector or empty vector were measured. Data are reported as means±SD for N=3 (ANOVA and *t*-test).

The transcription factor JDP2 overexpression significantly decreased the mRNA and protein expression of ITGBL1 in PC cells (Figure 6A and B). Using the Jaspar database, JDP2 was shown that it could bind to the promoter of ITGBL1. Moreover, results of the dual-luciferase assay showed that OE-JDP2 transfection significantly decreased the luciferase activity compared with the control vector upon transfection of the pGL3 vector containing ITGBL1 -2000 bp to + 50 bp fragments (Figure 6C). However, the deletion of -2000 bp to -1200 bp caused no significant change in ITGBL1 promoter activity in cells overexpressing JDP2, indicating that the core promoter region might be located within the range of -2000 bp to -1200 bp of ITGBL1 promoter. Considering the transcriptional regulation of ITGBL1 by JDP2, we further explored whether ITGBL1 mediated the effects of JDP2 on PC development. It was shown that ITGBL1 overexpression significantly increased the proliferation, migration, and invasion in JDP2-overexpressed cells (Figure 6D and E).

### ITGBL1 silencing suppressed tumor growth *in vivo*

**Figure 7:**
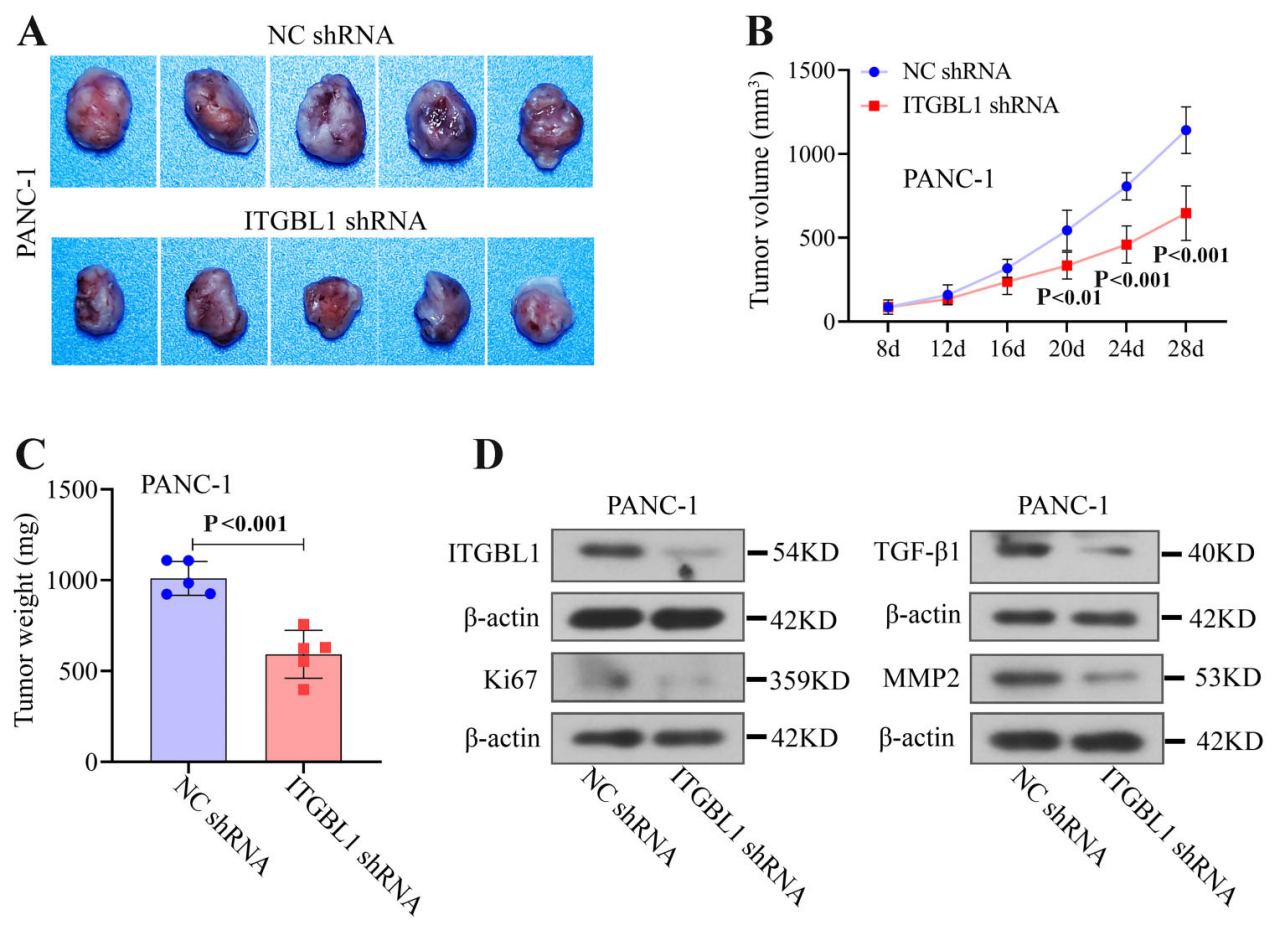
ITGBL1 silencing suppressed tumor growth *in vivo.* **A**-**C**, Tumor images, volumes, and weights of mice injected with ITGBL1-silenced cells or its control (NC). **D**, Protein expression levels of ITGBL1, Ki67, TGF-*β*1, and MMP2 in tumor tissues. Data are reported as means±SD for N=5 (ANOVA and *t*-test).

To further prove the ITGBL1's influence on the development of PC, ITGBL1-silenced PANC-1 cells or its control cells were injected into mice. The results showed that after injection of ITGBL1-silenced PANC-1 cells, tumor volumes of mice were decreased over time (Figure 7A and B). Furthermore, after injecting for 4 weeks, the tumor weights of mice injected with ITGBL1-silenced cells were significantly decreased (Figure 7C). Injection of ITGBL1-silenced cells also caused a significant decrease in ITGBL1 protein level in tumor tissues (Figure 7D). Ki67 (the typical proliferation marker), TGF-*β*1 (the TGF-*β*/Smad pathway-related factor), and MMP2 (metastasis-related factor) protein expressions in tumor tissues were also decreased by ITGBL1 knockdown (Figure 7D).

## Discussion

As the most persistent and deadly disease worldwide, PC has an aggressive course, low therapy efficacy, and poor prognosis (7,13). Moreover, current treatment strategies, including surgical resection, radiation, and chemotherapy, fail to cure PC (14). Therefore, it is of great significance to study potential therapeutic targets for the treatment of PC. In the present study, we mainly investigated the function and mechanism of ITGBL1 in PC. We found that ITGBL1 was highly expressed in PC tissues and the prognosis of PC patients with ITGBL1 high expression was poor (data from GEPIA database). PC cells with ITGBL1 knockdown exhibited decreased proliferation, migration, and invasion abilities, whereas ITGBL1 overexpression reversed these malignant behaviors. Moreover, ITGBL1 could activate the TGF-*β*/Smad pathway and its expression was transcriptionally suppressed by transcription factor JDP2, indicating that ITGBL1 may be a therapeutic target for the treatment of PC.

Previous studies have demonstrated that ITGBL1 is highly expressed in some types of cancer and its overexpression promotes cancer progression in colorectal cancer (15), ovarian cancer (16), and hepatocellular carcinoma (17). However, Gan et al. (18) demonstrated that ITGBL1 may be a tumor suppressor in non-small cell lung cancer (NSCLC). Downregulated ITGBL1 predicted a poor prognosis of patients with NSCLC and ITGBL1 overexpression inhibited the migration and invasion of NSCLC cells (18). The above findings suggest that ITGBL1 functions differently in different types of cancer. In the present study, we found that ITGBL1 was upregulated in PC tissues and overexpression of ITGBL1 stimulated the malignant progression of PC, such as proliferation, migration, and invasion. Similarly, the function of ITGBL1 in gastric cancer has been shown by Li et al. (11), which identifies ITGBL1 as a potential biomarker for the prognosis of patients with gastric cancer. Furthermore, we demonstrated that ITGBL1 could activate the TGF-*β*/Smad pathway in PC cells. The TGF-*β* signaling pathway has been proven to play important roles in various biological processes, including cell growth, differentiation, apoptosis, migration, as well as cancer initiation and progression (19). Also, the activation of TGF-*β* signaling is observed in PC and its blockade inhibits PC progression (20). The present study demonstrates that ITGBL1 may promote the development of PC through the TGF-*β*1/Smad pathway. Meanwhile, ITGBL1 promotes the proliferation of gastric cancer cells through activating the Akt pathway, which is also a positive regulatory pathway to promote the malignant progression of PC (21,22). In addition, ITGBL1 promotes invasion and migration by activating NF-κB signaling pathway in prostate cancer, and the activation of NF-κB signaling pathway also promotes PC progression (10,23). Those findings indicate that ITGBL1 may participate in the development of pancreatic cancer through multiple pathways.

JDP2, as a bona fide member of the activator protein-1 (AP-1) family, is deemed to be one of the combined proteins with c-Jun (24). AP-1 has been regarded as mediating the regulation of cellular processes, such as proliferation, apoptosis, and differentiation (25). It is has been reported that JDP2 plays an important role in cancer development and progression. For instance, JDP2 has been shown to strengthen the proliferative response and inflammation of the liver cancer model (26). Furthermore, JDP2 is relevant to tumor progression and poor prognosis in patients with pancreatic carcinoma (27). In addition, JDP2 can impede the epithelial-to-mesenchymal transition in PC (28). These findings solidly suggest that JDP2 can not only influence the modulation in other cancers but also inhibit the development of PC. Herein, we demonstrated that JDP2 could bind to the promoter of the *ITGBL1* gene and suppressed its transcription. Moreover, ITGBL1 overexpression reversed JDP2-mediated cell function in PC, which indicates that ITGBL1 may mediate the regulation of JDP2 on PC. Additionally, Li et al. (29) have shown that ITGBL1, as a Runx2 transcriptional target, facilitates breast cancer bone metastasis by stimulating the TGF-*β* signaling pathway. Furthermore, other genes are regulated by JDP2, such as transcription factor 3 (30), suggesting that JDP2/ITGBL1 axis may be one of the ways to participate in the malignant development of PC.

### Conclusions

In summary, our study indicated that ITGBL1 was up-regulated in PC tissues and promoted PC cell proliferation, migration, and invasion via the TGF-*β*/Smad pathway. Furthermore, we demonstrated that ITGBL1 expression was inhibited by transcription factor JDP2 by promoter binding. Our findings may provide a new direction in molecular-targeted therapy for PC.
